# A Numerical Study on the Effect of Debris Layer on Fretting Wear

**DOI:** 10.3390/ma9070597

**Published:** 2016-07-20

**Authors:** Tongyan Yue, Magd Abdel Wahab

**Affiliations:** 1 Department of Electrical Energy, Systems and Automation, Faculty of Engineering and Architecture, Ghent University, Zwijnaarde B-9052, Belgium; tongyan.yue@ugent.be; 2 Division of Computational Mechanics, Ton Duc Thang University, Ho Chi Minh City, Vietnam; 3 Faculty of Civil Engineering, Ton Duc Thang University, Ho Chi Minh City, Vietnam; 4 Soete Laboratory, Faculty of Engineering and Architecture, Ghent University, Technologiepark Zwijnaarde 903, Zwijnaarde B-9052, Belgium

**Keywords:** fretting wear, debris layer, finite element analysis (FEA)

## Abstract

Fretting wear is the material damage of two contact surfaces caused by micro relative displacement. Its characteristic is that debris is trapped on the contact surfaces. Depending on the material properties, the shapes of the debris, and the dominant wear mechanisms, debris can play different roles that either protect or harm interfaces. Due to the micro scale of the debris, it is difficult to obtain instantaneous information and investigate debris behavior in experiments. The Finite Element Method (FEM) has been used to model the process of fretting wear and calculate contact variables, such as contact stress and relative slip during the fretting wear process. In this research, a 2D fretting wear model with a debris layer was developed to investigate the influence of debris on fretting wear. Effects of different factors such as thickness of the debris layer, Young’s modulus of the debris layer, and the time of importing the layer into the FE model were considered in this study. Based on FE results, here we report that: (a) the effect of Young’s modulus of the debris layer on the contact pressure is not significant; (b) the contact pressure between the debris layer and the flat specimen decreases with increasing thickness of the layer and (c) by importing the debris layer in different fretting wear cycles, the debris layer shows different roles in the wear process. At the beginning of the wear cycle, the debris layer protects the contact surfaces of the first bodies (cylindrical pad and flat specimen). However, in the final cycle, the wear volumes of the debris layers exhibit slightly higher damage compared to the model without the debris layer in all considered cases.

## 1. Introduction

Fretting, in the tribology field, is a small oscillatory motion between contact surfaces. Fretting wear is a wear phenomenon caused by fretting [1], which is the dominant fretting damage in a gross sliding condition. This damage, in a practical application, may happen in every contact surface suffering from a time variable load. For example, in the stem/cement of a hip joint contact, fretting wear could cause aseptic loosening of the femoral component [2,3,4]. In the blade/disk of a dovetail joint in a turbine, it may increase the maintenance cost [5,6] and when it occurs in the interface between wires of steel ropes, it shortens the service life owing to decreasing the wire diameter [7,8]. Furthermore, if fretting wear takes place between the surfaces of electrical connectors [9,10], the contact resistance will increase sharply due to the oxide debris covering on the contact surfaces. 

In evaluating damage due to fretting wear, debris generated during the fretting wear process plays an important role. One characteristic of fretting wear is that a part of the contact surface is never exposed to the surrounded environment, due to the micro relative displacement. Thus, the debris has little opportunity to escape from the interface [11]. In such a case, the debris participates in the process of fretting wear and plays different roles. It can protect or harm interfaces based on material properties, sharpness of debris, and dominant wear mechanisms [12]. Due to this complicated character, researchers have focused on debris to study the process of fretting wear. In such a case, the Archard model proposed by Archard in 1953 [13], which is widely used for wear calculation and simulation, is not suitable since it is based on contact mechanics ignoring the real damage process of wear in practice. In 1973, a delamination theory of wear was proposed by Suh [14]. Compared with the Archard model, one advantage of this theory is that the actual micro-mechanism based on failure and damage processes is taken into consideration, which is closer to the practical situation. This model predicted that the debris shape was likely to be of thin flake-like sheets and could undergo large plastic deformation. One year later, this was confirmed by Waterhouse and Taylor [15]. They studied fretted surfaces of carbon steel, pure titanium, and Al-Zn-Mg alloy and found that loose wear debris caused by the propagation of sub-surface cracks was similar to that postulated in the delamination theory of wear. Furthermore, in a relatively recent research [12], it was found that the surface hardness of steel had an impact on the debris retention in the fretting wear process. Even for the same fretting coupling studied by K. Elleuch et al. [16], the form and component of debris were related to the displacement amplitude threshold. 

Due to the complicated role of debris in fretting wear, some researchers developed finite element (FE) models of debris during the fretting wear process. Researchers at Nottingham university modified the fretting wear FE model in Reference [17], to simulate the debris layer accumulated on the contact surfaces [18,19]. In this simulation tool, debris effects on wear damage were studied based on the Archard wear model and oxidation model, by redistributing the contact pressure and relative slip between the contact surfaces. Basseville et al. [20] proposed a fretting wear model, which explicitly included rectangular particles of a fixed number to simulate the debris. Benjamin et al. [21] developed a combined finite-discrete element tool for fretting wear modelling with the effect of debris. Although significant progress has been achieved in modelling the debris in the wear process, improvement would be realized in the mechanical property definition of debris, the contact property between debris and substrate, by modelling the evolution behavior of debris etc. More recently, an investigation of debris particles on fretting contact in partial slip was presented by Ghosh et al. [22]. They studied the material properties, i.e., elastic plastic deformation, and effect of the number of debris particles on contact and fretting behavior. They found that the debris particles were plastically deformed and underwent a significant part of the load applied on the body. In addition, they proposed that the fretting wear had no direct relation with the number of debris particles. Meanwhile, some assumptions have beene made for simplification, e.g., neglecting the evolution of fretting wear scar and keeping the stick zone size constant during the wear process.

The motivation of this work is to develop an FE model in order to improve the prediction of the experimental results and to provide a deep inside into the behavior/influence of debris in the fretting wear phenomenon. This paper is divided into four parts. After the introduction section, the FE model is described. Then, the effect of the debris layer is investigated and discussed. Finally, the conclusion is presented.

## 2. FE Model Description

**Figure 1:**
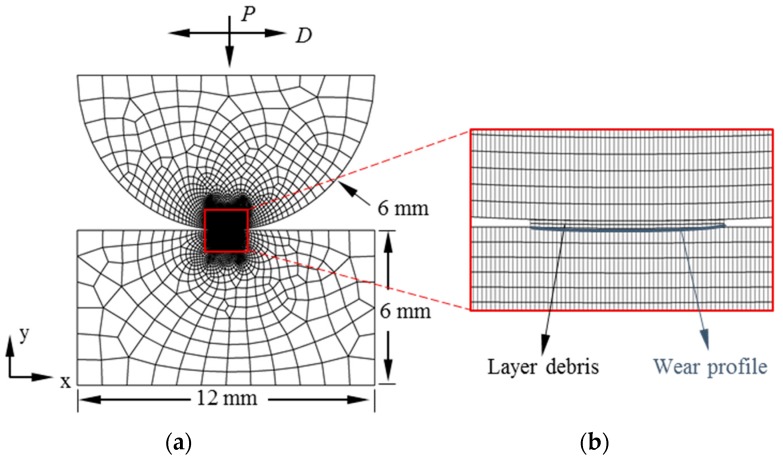
The fretting wear model with debris layer: (**a**) global scale with dimensions and (**b**) local configuration at the contact zone.

In this paper, a 2D plane strain cylinder/flat FE fretting wear model with a debris layer (named debris layer model) was constructed to study its effects on the wear profile. A model without a debris layer was presented in our previous work [23]. Global dimensions of the model used herein are as follows: the radius of the cylindrical pad is 6 mm and the specimen is 12 mm × 6 mm, as illustrated in Figure 1. Fretting wear loads are realized in two steps. Firstly, a constant normal load is applied at the mid-point of the top surface on the pad. Only the vertical motion of the pad is allowed at this step. Next, the horizontal displacement is imposed at the same point as the normal load is applied. The movement of the specimen is fully constrained during the fretting wear simulation. A plane strain 4-node bilinear element is used in the first bodies (pad and specimen). The mesh size of the contact surface in the first bodies is 5 µm.

**Figure 2:**
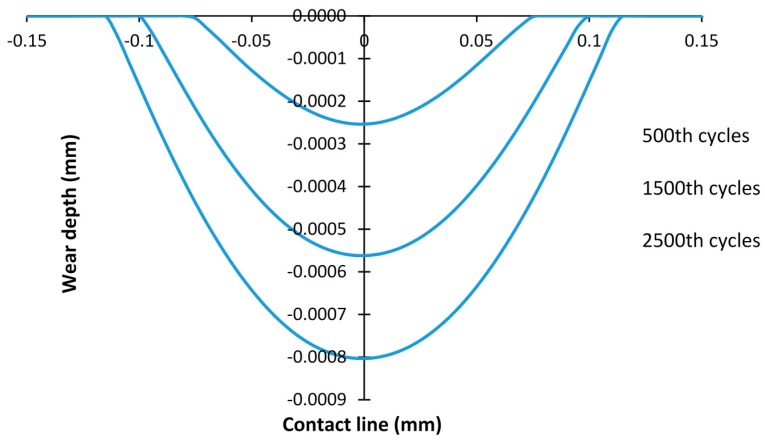
Wear scars of the specimen after different fretting wear cycles; *P* = 185 N and *D* = 25 µm.

The profile of the debris layer is defined by the wear scar calculated from the same fretting wear model without the debris layer (basic model). Fretting wear simulation of the basic model was presented in our previous work [23]. In this research, the debris layer model was generated by importing the layer with the same profile as the relevant wear scar of the specimen after 500, 1500, and 2500 fretting wear cycles, respectively. Under the loading condition of *P* =185 N and *D* = 25 µm, it is in a gross sliding regime. Figure 2 shows surface profiles of the specimen after 500, 1500, and 2500 cycles. The thickness of the layer is not kept constant during the fretting wear; therefore, the effect of thickness is also studied here. In this study, the thicknesses of layer *t* = 5 µm, 10 µm, and 20 µm are considered. The mesh size for the layer is 5 µm × 2.5 µm for *t* = 5 µm, and 5 µm × 5 µm for the other two thickness cases, respectively. The same type of elements as for the first bodies is employed for the debris layer.

**Figure 3:**
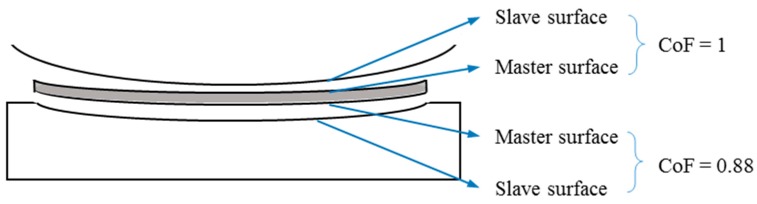
Contact interaction of the debris layer model.

In this debris layer model, two contact interfaces were created: (a) the contact surface between the pad and the debris layer and (b) the contact surface between the debris layer and the specimen. It is difficult to obtain the Coefficient of Friction (CoF) between these two interfaces separately by experimental measurements. However, CoF is also one of the important parameters to determine fretting behavior and wear extent. In this study, the CoF between the pad/layer is assumed to be equal to 1 and the CoF between the layer/specimen is taken as 0.88 based on the experimental data from Reference [17]. Usually, the debris of metallic fretting wear is a metallic oxide that is harder than the first bodies, therefore the top and bottom surfaces of the layer are defined as master surfaces for the pad/layer contact interface and the layer/specimen interface, respectively. These descriptions for the contact interfaces are also shown in Figure 3. For both interfaces, tangential behavior is constrained by the Lagrange multiplier method.

Several assumptions are made in this debris layer model:

- Only elastic deformation of the debris layer is considered. However, the elastic-perfectly-plastic deformation is taken into account for the first bodies.
- The layer covers the whole worn profile. Therefore, the width of the layer changes based on the wear width of the wear scar. The thickness of the layer in a given layer model does not change during wear simulation.
- The debris layer is homogeneous.

The material of the first bodies is a high strength alloy steel, the same as the one used in Reference [17]. The debris layer material is $\alpha -{Fe}_{2}O_{3}$ for the fretting wear of steel based on the experimental results of Reference [23]. The material property of this oxidation is cited from [24] and listed in Table 1.

**Figure 4:**
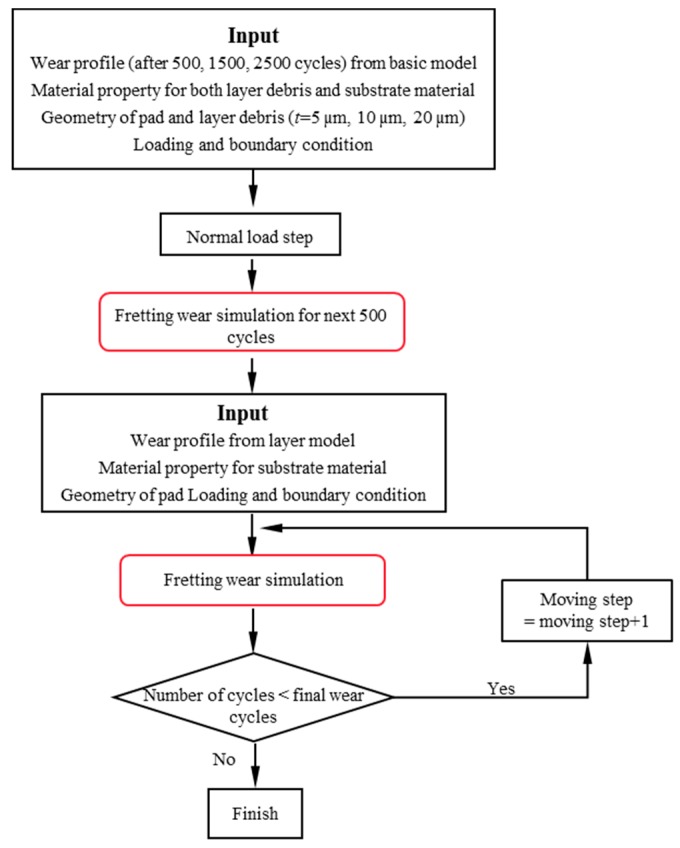
The flowchart of the fretting wear simulation with the debris layer.

The fretting wear simulation with a debris layer follows the flowchart shown in Figure 4. The wear profile predicted by the basic model after a certain number of cycles is imported as the initial geometry in the debris layer model. Next, the constant normal load *P* = 185 N is applied at the mid-point of the top surface of the pad. In this step, only the vertical movement of the pad and layer are allowed, and the specimen is fully constrained except for the contact surface. Subsequently, a horizontal displacement with amplitude 25 µm is imposed at the same point as *P* to simulate fretting wear. Under this condition, the debris layer is restricted by the contact algorithm. The layer effect is simulated explicitly only for the next 500 wear cycles of the debris layer model and the influence of the layer is recorded in the evolution of the wear scar of the specimen. Therefore, the wear profile from the layer model is imported to the next wear simulation without the layer. Finally, the fretting wear is calculated with the jump cycle 1000 and 100 increments per jump cycle step, until the number of cycles reaches the final number of wear cycles, namely 18,000 cycles.

All models are calculated by the ABAQUS/Standard with FORTRAN subroutine Umeshmotion. In this study, fretting wear is restricted to the flat specimen. The energy wear coefficient is 3.33 × 10^−8^ MPa^−1^ taken from [25]. The wear calculation is based on the energy concept proposed by Fouvry in 1996 [26]. 

## 3. Results

### 3.1. Contact Pressure Distribution before the Fretting Wear Process

At different time stages, the composition of the debris varies from metallic debris to oxide. Meanwhile, the thickness of the debris layer also changes with the number of fretting wear cycles. Therefore, before calculating the wear, the influences of the debris layer *E* and the thickness of layer *t* on the contact pressure are studied. Both interfaces of contact, i.e., the interface between pad/layer (top) and layer/specimen (bottom), are studied. In this case, only the normal loading step is considered. Young’s modulus used herein is in the range between 120 GPa and 360 GPa, i.e., it varies as 280, 200, and 120 GPa. The thickness of the debris layer is 5 µm, 10 µm, and 20 µm. The debris layer FE model with *E* = 360 GPa, *ν* = 0.12 and *t* = 5 µm is defined as a reference case. 

**Figure 5:**
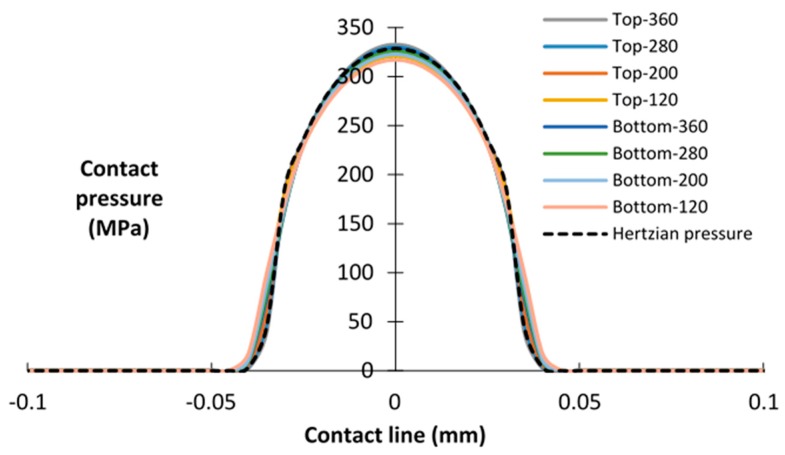
Contact pressure distributions of the top and bottom interfaces for different *E*s of the debris layer; *t* = 5 µm.

Figure 5 illustrates the contact pressure distributions in both top interface and bottom interface. In both interfaces, contact pressures are analogously distributed as Hertzian contact pressure. These contact pressures show little difference with different Young modulus *Es* values in both interfaces. A similar tendency of contact pressure was reported by Arnab et al. [22]. In their study, debris due to fretting wear was assumed as a sphere. By increasing Young’s modulus from 200 GPa to 400 GPa, no differences were shown on contact pressure distributions. This may be explained as most of the load is carried by first bodies, and the material properties of first bodies are identical in all simulations. Thus, this result indicates that the Young’s modulus of the debris layer has only a minor influence on the contact pressure distribution for both interfaces, when the thickness of the layer is kept constant, i.e., 5 µm. 

**Figure 6:**
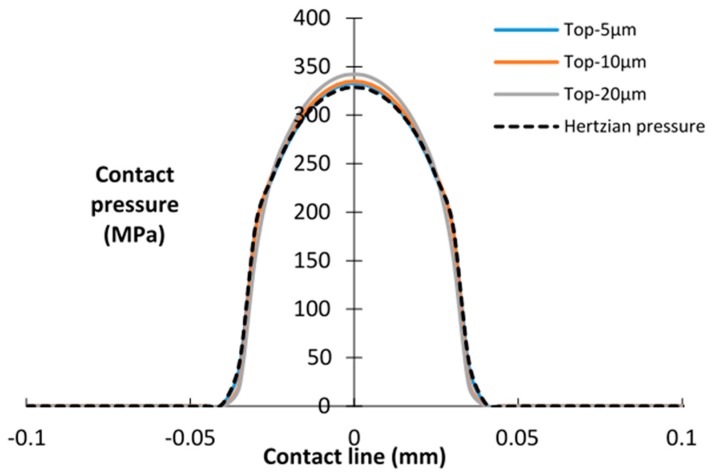
Contact pressure distributions of the top interface for different thicknesses of the debris layer; *E* = 360 GPa.

**Figure 7:**
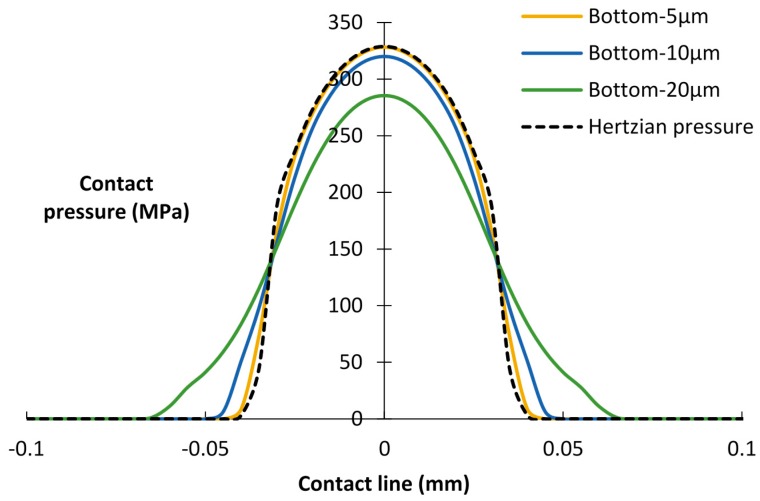
Contact pressure distributions of the bottom interface for different thicknesses of the debris layer, *E* = 360 GPa.

Next, the effects of the debris layer thickness on contact pressure were studied. As shown in Figure 6, little difference exists in the contact pressure distribution of the top interface. However, considering the bottom interface (Figure 7), the peak contact pressure decreases and the contact width increases with increasing thickness of the layers. It can be seen that a relatively thick layer carries more load and enlarges the contact area of the bottom interface. This tendency is also mentioned in the research of Reference [27].

Based on the contact pressure distribution in Figure 6 and Figure 7, it could be concluded that the contact pressure of the bottom interface is more sensitive to the thickness of the layer than the Young’s modulus of the layer. Given that fretting wear occurs at the bottom interface, in the following analysis, only the effects of the thickness of the debris layer are considered.

### 3.2. Contact Pressure Distribution during the Fretting Wear Process

**Figure 8:**
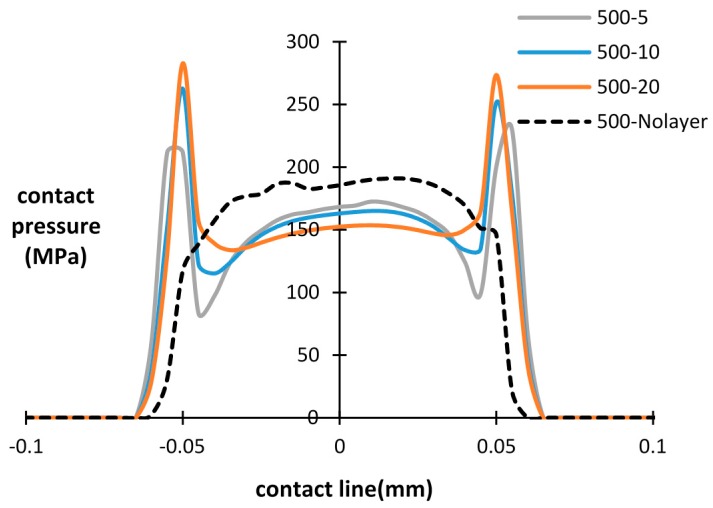
Contact pressure distribution for different layer thicknesses and number of cycles: layer thickness = 5 µm, 10 µm, 20 µm and without layer after 500 cycles.

**Figure 9:**
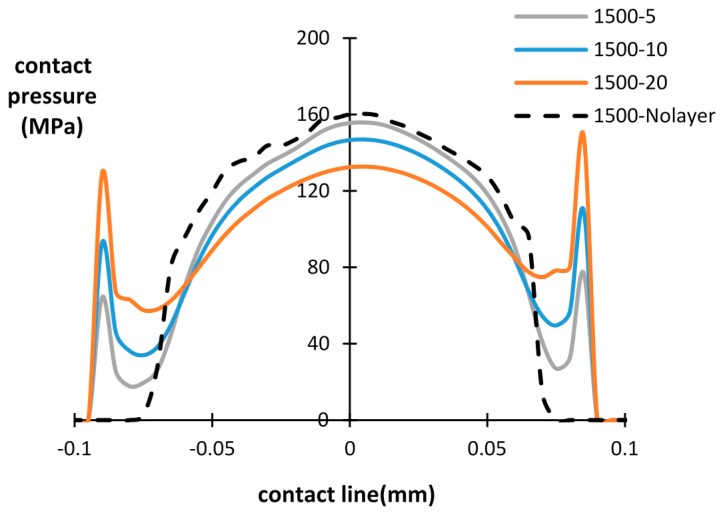
Contact pressure distribution for different layer thicknesses and number of cycles: layer thickness = 5 µm, 10 µm, 20 µm and without layer after 1500 cycles.

**Figure 10:**
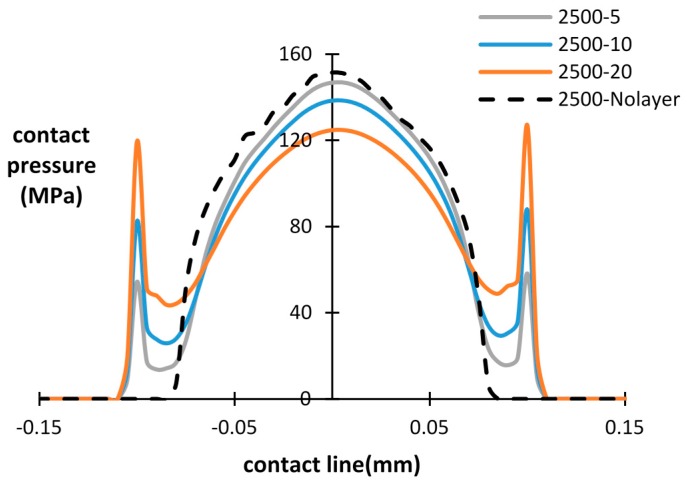
Contact pressure distribution for different layer thicknesses and number of cycles: layer thickness = 5 µm, 10 µm, 20 µm and without layer, after 2500 cycles.

The influence of the debris layer on contact pressure distribution during fretting wear cycles is investigated in this section. The contact pressure of the bottom interface after a different number of cycles is presented in Figure 8, Figure 9 and Figure 10, with different thicknesses of the debris layer. For all three importing time cases, a similar tendency of contact pressure with layer thickness is observed. It was found that after the same number of cycles, the contact pressure at the center of the specimen was reduced by increasing the thickness of layer. However, the contact pressure at the wear scar edge shows the opposite trend. The contact pressure dramatically increases at the wear scar edge due to the stress concentration at the layer edge. This is a significant difference from the contact pressure distribution of the basic model, in which case the contact pressure decreases continuously to zero at the contact edge. Similar results reported in research [18,22] show that the predicted peak contact pressure shifts to the contact edge due to the introduction of the debris layer and debris particles, respectively.

By increasing the number of fretting wear cycles, the contact pressure is reduced and the contact width increases due to the evolution of the contact surface in both the with/without debris layer models. For the debris layer model, the location of the peak contact pressure is shifted to the layer edge after 500 cycles for all three thicknesses. For the later importing time cases, i.e., after 1500 cycles and 2500 cycles, however, the maximum contact pressure is shifted to the central point of the contact surface in the lower thickness cases, i.e., *t* = 5 µm and 10 µm. For the case of *t* = 20 µm, the contact pressure at the layer edge also reduces dramatically from around 282 MPa to 150 MPa, which is similar to that of the central point in the contact.

Based on Figure 8, Figure 9 and Figure 10, it is clearly seen that the introduced debris layer significantly affects the distribution of the contact pressure, as well as, its magnitude. Generally, the debris layer carries a portion of the load and induces stress concentration at the debris layer edge. Thus, the wear scar could vary due to the introduction of the debris layer comparing with the model without the debris layer.

### 3.3. Wear Scar Comparison after Importing the Debris Layer

**Figure 11:**
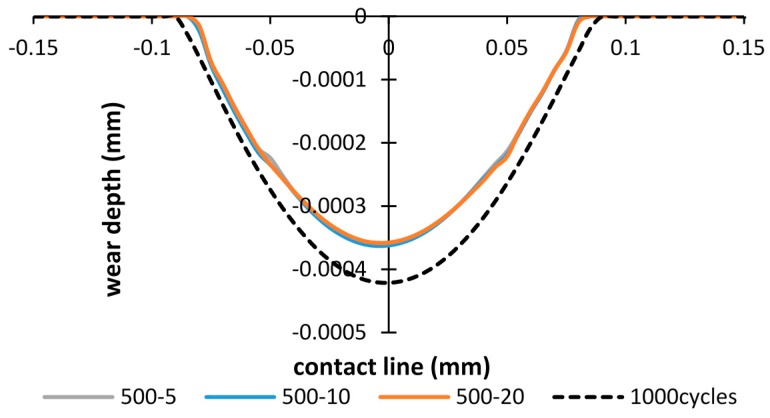
Wear scars after 1000 cycles: importing time= 500 cycles and layer thickness = 5 µm, 10 µm, 20 µm.

In order to study the effects of the debris layer on the wear scar, firstly, the wear scars after importing the debris layer by 500 cycles of fretting are compared. Figure 11 shows wear scars of the debris layer model in the case of importing time of 500 cycles and total number of cycles of 1000 cycles. It was found that wear scars are almost the same in all three thickness cases. Thus, the effect of thickness on the wear scar is minor. Also, comparing with the basic model, both wear width and wear depth calculated by the debris layer model reduce to 5% and 15%, respectively. 

**Figure 12:**
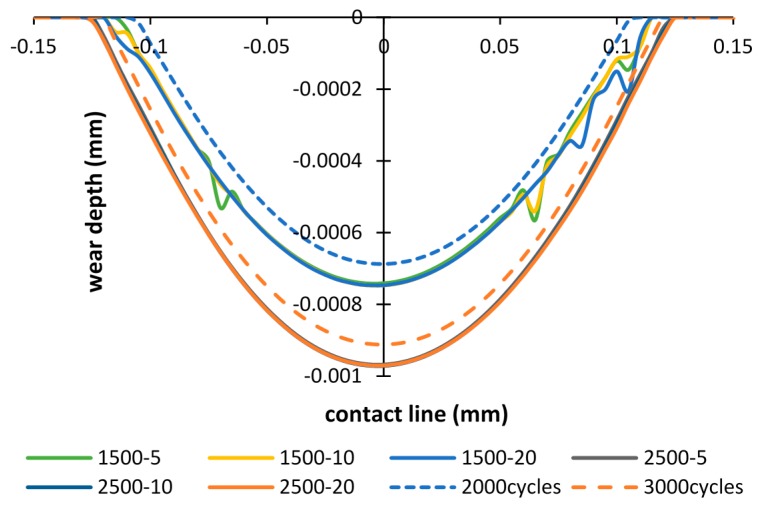
Wear scars after 2000 cycles and 3000 cycles: importing time = 1500, 2500 cycles and layer thickness = 5 µm, 10 µm, 20 µm.

However, in the cases: importing time = 1500 and 2500 cycles (Figure 12), the opposite tendency is shown, i.e., more wear damage is generated in both cases. The wear depth increases by 8% and 6%, after 2000 cycles and 3000 cycles, respectively. Whereas, the wear width increases by 4.8% and 4.1% after 2000 cycles and 3000 cycles, respectively. 

### 3.4. Wear Scar after Running-in Stage

**Figure 13:**
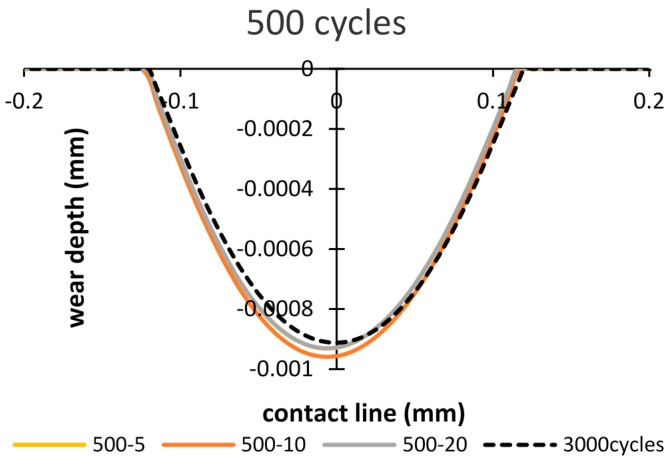
Wear scar after 3000 cycles of the debris layer model: importing time = 500 cycles and layer thickness = 5 µm, 10 µm, 20 µm.

**Figure 14:**
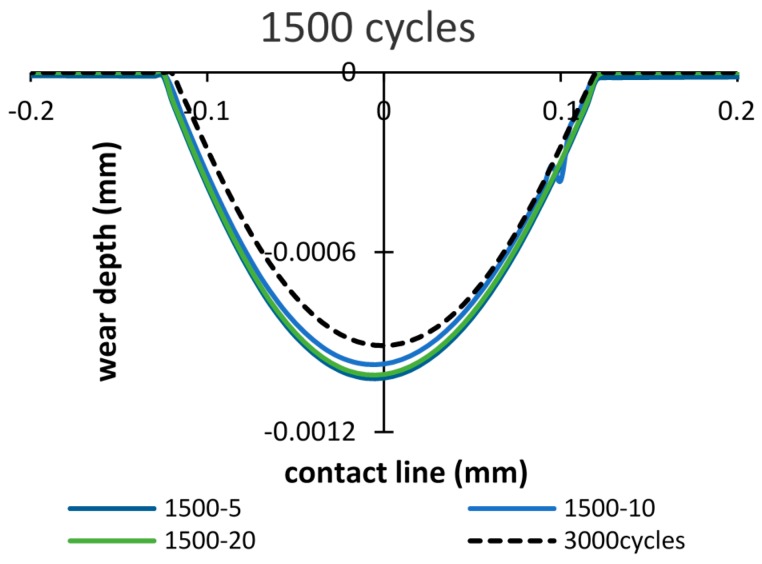
Wear scar after 3000 cycles of the debris layer model: importing time = 1500 cycles and layer thickness = 5 µm, 10 µm, 20 µm.

**Figure 15:**
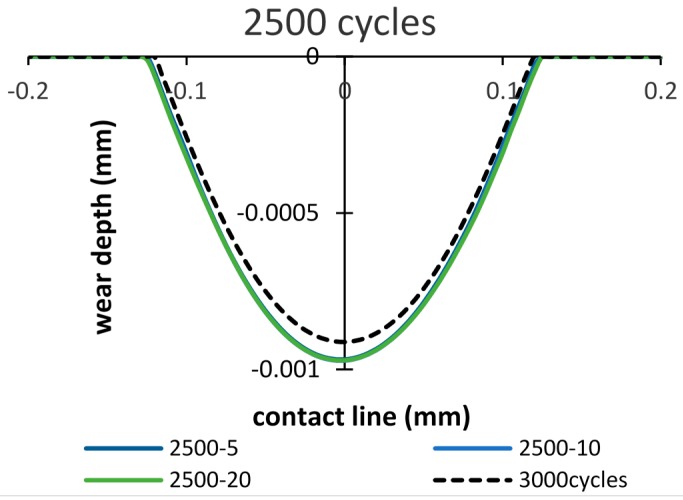
Wear scar after 3000 cycles of the debris layer model: importing time = 2500 cycles and layer thickness = 5 µm, 10 µm, 20 µm.

The wear scars after the running-in stage of 3000 cycles are compared in Figure 13, Figure 14 and Figure 15. Figure 13 shows different wear scars when the importing time is 500 cycles. The influence of thicknesses has little effect on the wear scar, and is approximately similar to the results of the basic model.

When importing the debris layer in later cycles, the differences between the wear scars obtained from models of with and without the debris layer still could be seen, as shown in Figure 14 and Figure 15. However, the differences in wear depth and wear width are decreased compared to those after 2000 and 3000 cycles, respectively. 

### 3.5. Final Wear Scar

**Figure 16:**
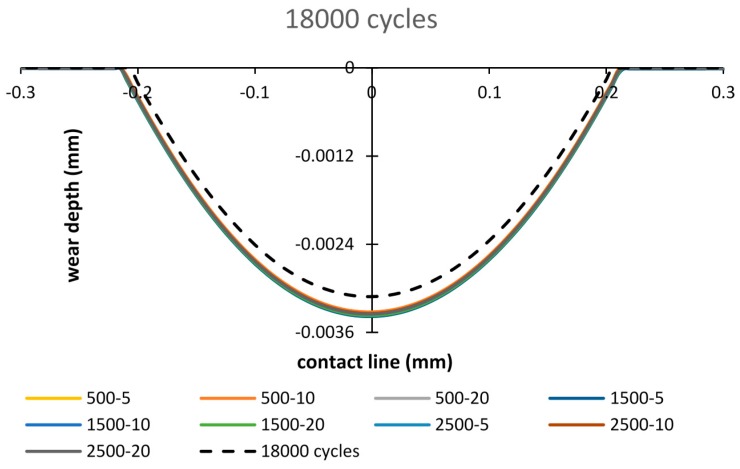
Wear scar after 18,000 cycles based on debris layer model: importing time = 500, 1500, 2500 cycles and layer thickness = 5 µm, 10 µm, 20 µm.

The final wear scars after 18,000 cycles are presented in Figure 16. Minor differences are observed among FE results of different debris layer models. However, compared to the case of the basic model, introducing the debris layer induces more wear damage.

## 4. Discussion

This research focuses on the role debris plays during the fretting wear process. In this study, debris is assumed as the layer structure of $\alpha -{Fe}_{2}O_{3}$ with three thicknesses, i.e., 5 µm, 10 µm, and 20 µm. The debris layers were imported after different cycles in the running-stage. Based on this debris layer model, the wear damage after importing debris, after the running-in stage and the final cycle were compared. The wear damage was calculated as the wear volume, i.e., integration of the scar area and the wear depth.

**Figure 17:**
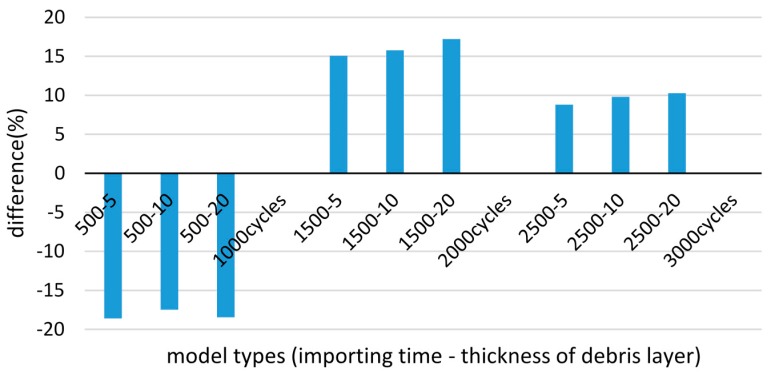
Difference of wear damage compared to the case of basic model, after 1000 cycles, 2000 cycles and 3000 cycles.

Among the three importing time stages, as shown in Figure 17, the wear damage of 500 cycles was underestimated by approximately 18% comparing to the basic model. As explained in Section 3.2, with the introduction of the debris layer, the contact pressure distribution is changed so that stress concentration also exists at the contact edge of the debris and substrate surface, beside the contact center. Thus, following the Coulomb friction law, the shear stress distribution also shifts. In addition, after 500 cycles, the contact width, in the models with and without debris layer, are similar due to the micro scale wear damage at the beginning stage of fretting wear. In this case, the reduction of shear stress causes decreasing wear damage after 1000 cycles. In contrast, the wear damages after 1500 cycles and 2500 cycles are all higher than in cases without layers. This could be explained by the combination of contact pressure and contact width. On increasing the number of fretting cycles, the wear width becomes relatively larger as the length of the debris layer increases. The contact width enlarges the edge of the debris layer, which is much wider than in the case without debris layer. Hence, although the shear stress is still less than in the case without debris layer, the wear damage is severer according to the calculation based on the energy concept.

**Figure 18:**
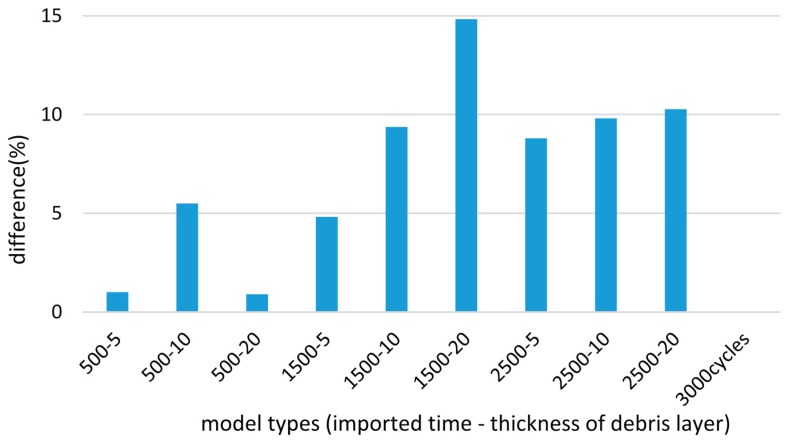
Difference of wear damage compared to the case of basic model, after 3000 cycles.

**Figure 19:**
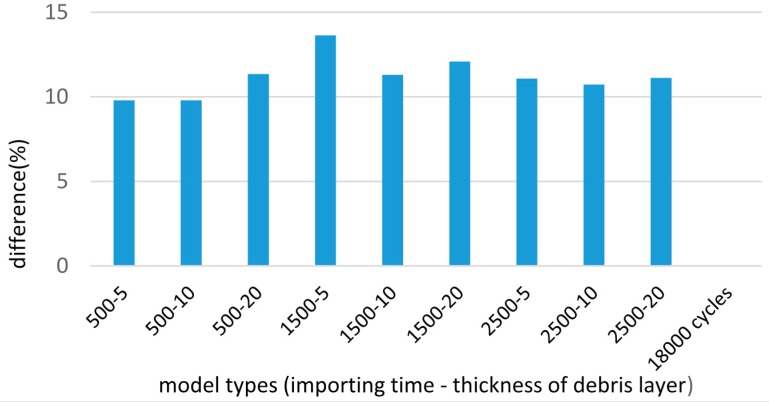
Difference of wear damage compared to the case of basic model, after 18,000 cycles.

After the running-in stage, more wear damage occurs in most cases of the debris layer model (Figure 18). For the final cycles, the appearance of the debris layer brings more wear damage by 10% to 13.6% depending on the importing time and the thickness of the debris layer (Figure 19).

Based on the evolution of wear damage, it can be seen that the debris layer is a protection of the first bodies at the beginning of fretting wear. With increasing number of cycles, the wear damage grows quickly due to the enlarged contact area, and more wear damage occurs compared to the model without debris layer. However, in the study of J. Ding [18], the wear volume predicted by the debris model was less than in the case without debris layer and was underestimated compared to the experiment results. In addition, the introduction of the debris layer induced smaller scar width and slightly deeper wear depth, which is also different from the results of the debris model presented here. The possible reasons are:

- The material models of the debris layers used are different. The material behavior of the debris layer in reference [18] was assumed to be anisotropic elastic-plastic. While with the material behavior presented here, it is assumed the debris layer is elastic.
- The assumption of contact involved debris layer is different. In reference [18], the contact of the top interface (between cylindrical pad and debris layer) is assumed to be a rigid connection. However, in this study, both top and bottom interfaces of contacts were implemented contacts.

Indeed, the debris model developed herein is based on several assumptions. Due to the complicity of the debris behavior in fretting wear, a more accurate debris model could be generated, e.g., multiscale modelling to simulate the motion of nano-scaled debris. In addition, the evolution of the thickness is difficult to predict. As reported in [23], the thickness of the layer did not increase monotonically with the number of cycles. More experiments should be done to study debris layer behavior in the fretting wear process.

## 5. Conclusions

A plane strain fretting wear model with a debris layer was developed to investigate the effects of debris on fretting wear damage. Both the Young’s modulus and the thickness of the debris layer were variable in this study. Meanwhile, the importing time of the layer was also introduced. Based on FE results, the major conclusions of this study are as follows:

- The contact pressure distribution before implementing fretting wear shows that Young’s modulus of the debris has little influence on the contact pressure in both top and bottom interfaces. However, the thickness of the layer plays an important role at the bottom interface. On increasing the thickness of the debris layer, the contact pressure is reduced and the contact area grows at the bottom surface.
- By importing the debris layer in different fretting wear cycles, the distribution of the contact pressure changes significantly. Stress concentration exists at the debris layer edges. At the early stage of fretting wear, the maximum contact pressure shifts from the central contact point to the debris layer edge.
- Comparing fretting wear scar, a minor difference could be found among different thicknesses of debris layer cases.
- At the beginning of fretting wear cycles, the debris layer protects the contact surface and reduces the wear volume compared to results from the model without the debris layer. While after 18,000 cycles, more wear damage occurs when considering the effects of the debris layer.

## Figures and Tables

**Table 1:** Material properties used in finite element (FE) models.

Material Properties	Values
Young’s modulus of first bodies (GPa)	200
Yield strength of first bodies (MPa)	1240
Young’s modulus of the debris layer (GPa)	360
Poisson ratio of first bodies	0.3
Poisson ratio of the debris layer	0.12
